# Community dynamics of microbial eukaryotes in intertidal mudflats in the hypertidal Bay of Fundy

**DOI:** 10.1038/s43705-023-00226-8

**Published:** 2023-03-14

**Authors:** Eke I. Kalu, Adrian Reyes-Prieto, Myriam A. Barbeau

**Affiliations:** Department of Biology, University of New Brunswick, Fredericton, NB Canada

**Keywords:** Microbial ecology, Biodiversity, Next-generation sequencing

## Abstract

Protists (microbial eukaryotes) are a critically important but understudied group of microorganisms. They are ubiquitous, represent most of the genetic and functional diversity among eukaryotes, and play essential roles in nutrient and energy cycling. Yet, protists remain a black box in marine sedimentary ecosystems like the intertidal mudflats in the Bay of Fundy. The harsh conditions of the intertidal zone and high energy nature of tides in the Bay of Fundy provide an ideal system for gaining insights into the major food web players, diversity patterns and potential structuring influences of protist communities. Our 18S rDNA metabarcoding study quantified seasonal variations and vertical stratification of protist communities in Bay of Fundy mudflat sediments. Three ‘SAR’ lineages were consistently dominant (in terms of abundance, richness, and prevalence), drove overall community dynamics and formed the core microbiome in sediments. They are Cercozoa (specifically thecate, benthic gliding forms), Bacillariophyta (mainly cosmopolitan, typically planktonic diatoms), and Dinophyceae (dominated by a toxigenic, bloom-forming species). Consumers were the dominant trophic functional group and were comprised mostly of eukaryvorous and bacterivorous Cercozoa, and omnivorous Ciliophora, while phototrophs were dominated by Bacillariophyta. The codominance of Apicomplexa (invertebrate parasites) and Syndiniales (protist parasites) in parasite assemblages, coupled with broader diversity patterns, highlighted the combined marine and terrestrial influences on microbial communities inhabiting intertidal sediments. Our findings, the most comprehensive in a hypertidal benthic system, suggest that synergistic interactions of both local and regional processes (notably benthic-pelagic coupling) may drive heterogenous microbial distribution in high-energy coastal systems.

## Introduction

Hypertides or tidal ranges >6 m are a rare natural phenomenon, and the largest ones occur in the Bay of Fundy on Canada’s East Coast [1]. The upper reaches of the Bay of Fundy are home to vast intertidal mudflats (ca. 512 km^2^) and marked by semidiurnal tides that can exceed 15 m [2]. These mudflats are silt-dominated and exhibit steep biotic and environmental gradients [3–6]. Microalgal biofilms at the mud surface are responsible for an estimated half of the primary production in parts of this region; in turn, an estimated half of the organic matter produced is remineralized and buried in hypoxic subsurface sediments [7, 8]. Thus, the sediment microbiome is able to create nutritious foraging habitats for their faunal co-residents, as well as for visitors to the mudflat—including most of the global population of Semipalmated Sandpipers (*Calidris pusilla*) during their annual migration [9]—ultimately affecting global-scale ecological processes. In mudflats, carbon sequestration, energy and nutrient export to adjacent ecosystems, and coastal protection from biofilm-mediated sediment stability, are presumably also dependent on microbial activity [10–14]. Yet, much of the diversity and dynamics of the microbial players in mudflats remain a black box.

Our study is specifically concerned with protists (microbial eukaryotes), a critically important but understudied group of microorganisms. Protists are ubiquitous in soil [15, 16], freshwater [17] and marine [18, 19] ecosystems, and represent most of the diversity among eukaryotes [20–22]. They are essential players in global biogeochemical cycles [23] and play important roles as primary producers, consumers, decomposers, and symbionts of most animals and plants [15, 24–26]. While diatoms and dinoflagellates usually dominate microalgal assemblages in coastal sedimentary ecosystems, predators like ciliates and cercozoans, and obligate parasites like apicomplexans, serve to establish key associations in the food web [24, 27]. Resolving the spatial and temporal scales at which these protists vary will help to better elucidate their structuring influences.

In the present paper, we quantified spatial change (among-site distribution and vertical stratification) and seasonal variation in the composition of protist communities in intertidal mudflats in the Bay of Fundy, considering both a taxonomic and trophic functional perspective. To achieve this, we examined four sediment depths at two mudflats, from June to October 2019, using a high-throughput 18S ribosomal RNA (rRNA) gene sequencing–based approach [28, 29]. We also investigated associations between protist communities and physico-chemical properties of the sediment environment. Our analyses detected heterogenous protist distribution and identified a core eukaryotic microbiome in the intertidal mudflats. We discuss the potential roles of local and regional factors, to explain the observed patterns of protist distribution. Our findings provide a baseline for future comprehensive investigation of the deterministic and stochastic drivers of microbial community dynamics in intertidal mudflats [30, 31]. This study is the first of its kind in the Bay of Fundy and contributes to our limited understanding of the spatio-seasonal dynamics of benthic protists in hypertidal systems.

## Methods

### Study sites and sample collection

**Fig. 1:**
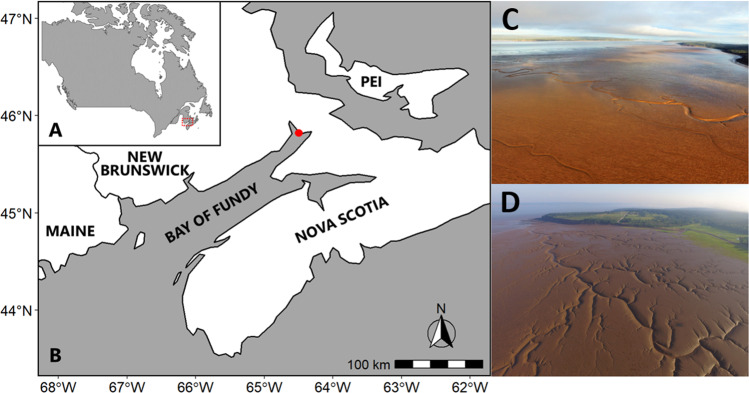
Study sites in the Bay of Fundy, Canada. Location of (**A**) the Bay of Fundy and (**B**) the study sites, Grande Anse and Pecks Cove, in the upper Bay of Fundy (red dot). Aerial views of the intertidal mudflat at both sites, with across-shore width of 2–3 km at (**C**) Grande Anse, and 0.8–1 km at (**D**) Pecks Cove. Photographs taken by G.S. Norris in summer 2019.

We sampled two mudflats: Grande Anse (latitude, longitude: 45.8078848, −64.4956354) and Pecks Cove (45.7522406, −64.4869961) (Fig. 1). These sites are representative of large (5–10 km along-shore by 1–2.5 km cross-shore), silt-dominated (average sediment particle sizes of 20–40 μm) intertidal mudflats in the upper Bay of Fundy, visited annually in mid to late summer by large flocks of migratory shorebirds [6, 9]. While just 6 km apart, our sites are in different arms of the Bay of Fundy and differ in sedimentary properties. Grande Anse sediments have smaller particle size, but greater penetrability, water content and organic matter content than Pecks Cove on average [6]. At each site, we established a study area 430 m long (along-shore) by 200 m wide, 300–500 m from shore at Grande Anse and 150–350 m from shore at Pecks Cove, so that both areas were at similar elevations (Fig. S1).

We sampled our sites five times from June to October 2019 (late spring to early fall) to correspond to natural disturbances on mudflats. They include (i) late spring, after winter ice scouring; (ii) early summer, (iii) mid summer, and (iv) late summer, before, during and after the stopover and associated intense foraging activity of migratory shorebirds [9], respectively; and (v) early fall, before the onset of overwintering temperatures (Table S1). We implemented a stratified random sampling design, as described by Norris et al. [32], for sample collection. We collected three replicate sediment cores per site and season, and a sampling location was never resampled. Sediment cores were collected using tip-less plastic syringes (10.6 cm long, 2.2 cm diameter) and stored at −80 °C until processing. Samples for environmental DNA isolation were later obtained from the top 5 mm of the core and the 5 mm sections around the 1 cm, 4 cm, and 7 cm depths. These four depths were chosen to correspond to sediments layers with different oxygen concentrations. They include (i) the surface microalgal biofilm layer; (ii) a few millimeters below the surface; (iii) near the maximum burrowing depth of most invertebrates, above which is periodically oxygenated by bio-irrigation; and (iv) within hypoxic sediments, near the permanently anoxic layer. We also measured sediment pH, redox potential, and temperature at each sampling location, at 1 cm increments from the sediment surface to a depth of 10 cm, using a portable meter and probe (models HI991003 and HI12973, respectively, Hanna Instruments, Woonsocket, Rhode Island, USA).

### Sequencing and analysis of the 18S rRNA barcode

Total environmental DNA was extracted from each sediment sample using the DNeasy PowerSoil Kit (Qiagen, Hilden, Germany) according to the manufacturer’s instructions. For each environmental DNA sample, PCR amplicons of the hypervariable V4 region of the 18S rRNA gene were generated (using primers E572F and E1009R) and sequenced on the Illumina MiSeq platform at the Integrated Microbiome Resource (Dalhousie University, Halifax, Canada), following reported protocols [33], thus generating 300-base paired-end reads.

We used QIIME 2 (Quantitative Insights Into Microbial Ecology, v2022.2) [34], implemented on the Microbiome Helper platform (v2.3.0) [35], for analysis of amplicon sequence data (Table S2). Sequence reads were quality-filtered (retention criterion: Phred score of ‘30’ over 90% of sequenced bases). Chimeric (mixed PCR product) and erroneous (resulting from sequencing error) reads were discarded, and amplicon sequence variants (ASVs, exact sequences) were clustered using Deblur and associated denoising tools [36]. Only ASVs with read counts ≥0.1% of the average number of reads per sample (i.e., non-rare ASVs) were retained and assigned taxonomic identities using a Naive-Bayes approach and the PR^2^ database (Protist Ribosomal Reference, v4.14.0) [37] as reference. ASVs assigned to animals, fungi, and land plants were discarded. Protist ASVs were retained and assigned to trophic functional groups and subgroups at the best taxonomic resolution attainable using the available comprehensive literature, including [21] and [38] (Table S3). Samples with low numbers of high-quality protist reads (i.e., <1000 reads retained from the bioinformatics process) were omitted from further analyses.

It is important to mention that the relationship between the copy number of the 18S rRNA gene and various cellular properties remains poorly understood. However, studies in certain protist lineages suggest that the number of gene copies more accurately reflects cellular biomass and biovolume than cell count [39–42]. Attempts at correcting 18S rDNA read abundance estimates for better approximations to cell counts are complicated by interspecific, strain-level and even geographic variations in the gene copy number [43]. As such, while lineage-specific correction factors have been proposed [44, 45], none are widely accepted or used. Here, we use the relative abundance of 18S rDNA reads as a proxy for relative cellular biomass.

### Statistical analyses

We evaluated how richness (number of observed ASVs) and composition (average percentage of reads for each taxon or trophic functional subgroup) of protist communities varied seasonally and with sediment depth at both sites using PRIMER (v6) [46] with the PERMANOVA add-on (Permutational Multivariate Analysis of Variance, v1.0.3) [47]. We analyzed Site (2 levels), Season (3 or 5 levels) and Depth (4 levels) as fixed factors, and Location (3 replicates, nested in Site and Season) as a random factor. PERMANOVAs were run using resemblance matrices generated with Euclidian distance (for richness) and the Bray–Curtis coefficient (for community composition) [46]. We estimated components of variation [47, 48] to assess the relative importance of factors and their interactions. PERMANOVAs were also run for the univariate read count (number of reads per sample) and multivariate sediment physico-chemical properties.

Given the strong site-level variation, and significant three-way interaction between the main factors (Site × Season × Depth), we conducted a separate PERMANOVA for each site, followed by planned contrasts focused on the effect of Depth. We conducted PERMDISP (Permutational Dispersion) tests to determine if significant PERMANOVA results were due to differences in centroids (multivariate averages) and/or differences in dispersion (multivariate variances) [47]. Finally, significant Depth effects were analyzed using SIMPER (Similarity Percentages) [49] to identify taxa or trophic functional subgroups contributing to differences in community composition.

To examine associations between (i) community composition (Bray–Curtis similarity matrices) and sediment physico-chemical properties (Euclidian distance similarity matrices on normalized data), as well as (ii) the taxonomic and trophic functional perspectives of community composition, we performed RELATE tests using Spearman’s rank correlation coefficient [46]. When significant associations were observed, we used the BEST (Biota-Environment matching + Stepwise) routine [46, 50] to identify sediment properties best correlated with variations in community composition.

## Results

Our sequencing effort, which recovered 562,941 quality-filtered 18S rDNA (V4 region) reads and 2118 ASVs of protists, was sufficient for richness to approach saturation at both local and global scales (Fig. S2). Average richness did not vary significantly among sites, seasons, or depths, except being significantly lower in early fall than other seasons at Grande Anse (Table S4). However, read count was far more dynamic (Table S5). The top 28 most abundant ASVs on average accounted for the majority of reads and were on average more prevalent (79% of samples) than the ASV pool (15%) (Table S3). Of the 2118 ASVs, 73% were present at both sites. 34% of ASVs were shared among all depths at Grande Anse, with 25% at Pecks Cove. Top contributors to the ASV pool were Cercozoa (43% of ASVs), Vampyrellida (10%), Apicomplexa (8%), and Bacillariophyta, Ciliophora and Dinophyceae (6% each).

### Spatio-seasonal variations in community composition

Cercozoa (37% of reads), Bacillariophyta (18%) and Dinophyceae (15%) were the most abundant taxa (Table S3). They comprised most protist reads on average (*n* = 93 samples), and we refer to them collectively as the ‘core’ taxa in sediments. Among Cercozoa, the classes Thecofilosea (60% of reads) and Imbricatea (29%) were most abundant. The order Cryomonadida comprised 61% of reads among Thecofilosea. Among Cryomonadida, the Protaspa-lineage (7% of protist reads and 100% prevalence) was dominant. On average, 71% of reads among Cercozoa were affiliated with testate cells, with organic theca eclipsing siliceous tests, while 13% were affiliated with naked flagellated cells. Most reads among Cercozoa were also affiliated with lineages known to engage in substrate gliding, as opposed to free swimming, for locomotion. Among Bacillariophyta, polar centric Mediophyceae comprised 58% of reads, followed by raphid pennates at 29%, araphid pennates at 11%, and radial centric basal Coscinodiscophyceae at 1.5%. *Thalassiosira* spp. (7% of protist reads and 100% prevalence) were dominant within polar centric Mediophyceae and Bacillariophyta more broadly. Among Dinophyceae, the orders Gonyaulacales (43% of reads) and Peridiniales (25%) were most abundant. The toxigenic, bloom-forming *Alexandrium fundyense* [51] (7% of protists reads and 90% prevalence) comprised the vast majority of Gonyaulacales.

Other taxa were much less abundant, with Ciliophora, Syndiniales, Chlorophyta, Apicomplexa, Vampyrellida, Apusomonadidae, and Amoebozoa each comprising, on average, 1–7% of protist reads (Table S3). Among Ciliophora, the classes Spirotrichea (53% of reads) and Phyllopharyngea (32%) dominated. Poorly resolved lineages of the order Strombidiida comprised most of Spirotrichea while the genus *Zosterodasys* (2% of protist reads) overwhelmingly dominated Phyllopharyngea. Syndiniales were almost entirely comprised of the Group I Clade 4 lineage (4% of protist reads). Likewise, among Apicomplexa, the class Gregarinomorphea (i.e., gregarines, 4% of protist reads) was exceedingly dominant, with archigregarines comprising 45% of reads. Chlorophyta was dominated by the classes Trebouxiophyceae (57% of reads) and Pyramimonadophyceae (specifically unresolved lineages of family Pycnococcaceae, 31%). The extremely productive and stress tolerant *Picochlorum* sp. [52, 53] (2% of protist reads, 56% of Chlorophyta reads and 100% prevalence) in the order Chlorellales, completely dominated Trebouxiophyceae.

**Fig. 2:**
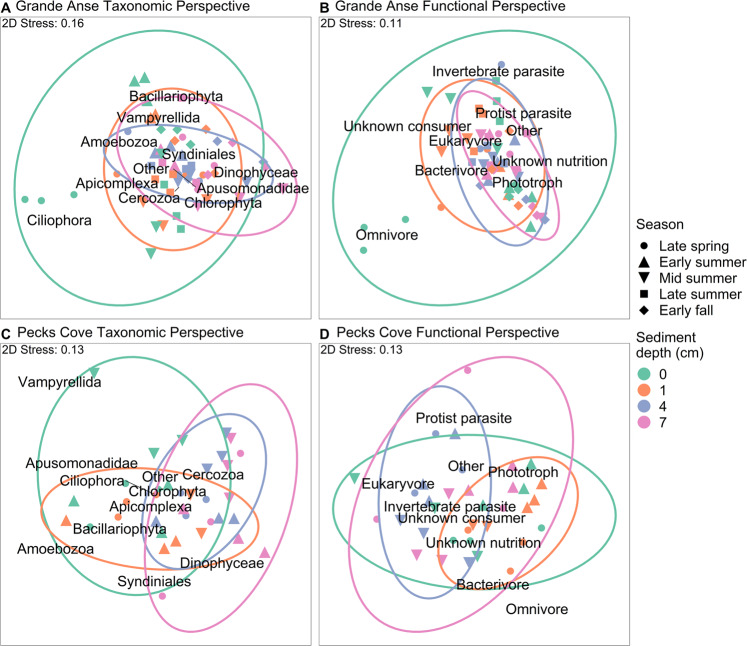
Non-metric multidimensional scaling (nMDS) graphs showing the relative placements of protist communities (a symbol represents a sample) in ordination space, for Bay of Fundy mudflats in 2019. Community dispersion around the centroid for each sediment depth, pooling over seasons, are captured by 95% confidence ellipses. Positions of taxa or trophic functional subgroups represent correlations with nMDS axes and indicate directions of increasing magnitude. “Other” comprises subgroups “Mixotroph”, “Vertebrate parasite”, “Unknown animal parasite”, “Plant parasite” and “Unknown parasite”, or taxa with <1% of average protist reads. Panels **A** and **C** illustrate the taxonomic perspective at each site, while **B** and **D** display the functional perspective. In **A** and **C**, the small lines indicate the actual placements of taxa in ordination space.

The taxonomic makeup of protist communities varied significantly among study sites, seasons, and sediment depths (i.e., the main effects) (Table 1). Significant two-way and three-way interactions of the main effects collectively comprised a third of the total spatio-seasonal variation in community composition. Community composition also varied substantially at our largest (Site, 27% of variation) and smallest (sample, 18%) spatial scales. Beyond their interactive effects, the factors Season and Depth each accounted for an intermediate amount of the total variation. These observed significant effects were a result of differences in centroids (multivariate averages of community composition) and not differences in the dispersion around centroids (Table 1). From the perspective of sediment depth, communities in our shallowest sediments (i.e., surface and 1 cm) significantly differed from one another, in contrast to those in our deepest sediments (i.e., 4 cm and 7 cm) which did not (Table 1). The taxonomic makeup of shallow and deep sediments was also significantly different. These depth patterns were consistent and recurrent at Pecks Cove, but seasonal at Grande Anse. At Grande Anse, seasonal variability in community composition peaked at the sediment surface (Fig. 2A) and variability among depths was generally larger in late spring (e.g., surface and 1 cm were 45% dissimilar) than other seasons (17–26% dissimilarity). As for Pecks Cove, community composition generally displayed a ‘depth decay’ trend (i.e., dissimilarity between depths increased with increasing vertical distance in sediments), as well as a ‘temporal decay’ pattern (Fig. 2C).

**Table 1:** PERMANOVA results for spatio-seasonal variations in protist taxa in Bay of Fundy mudflats in 2019.

Site	Source of variation	df	MS	Pseudo-*F*	*P*	Component of variation	
						Estimate	%
Both	Site	1	9 987	28.40	0.001	288	27.4
	Season	2	2 188	6.22	0.001	82	7.8
	Depth	3	1 912	9.87	0.001	103	9.8
	Site × Season	2	1 315	3.74	0.001	86	8.2
	Site × Depth	3	663	3.42	0.001	56	5.4
	Season × Depth	6	824	4.25	0.001	113	10.8
	Site × Season × Depth	6	433	2.23	**0.002**	86	8.2
	Location(Site × Season)	12	353			42	4.0
	Depth × Location(Site × Season)	33	194			194	18.4
Grande Anse	Season	4	2 757	12.97	0.001	223	28.0
	Depth	3	1 712	5.82	0.001	101	12.7
	Season × Depth	12	798	2.71	**0.001**	176	22.2
	Surface vs 1 cm	4	791	3.29	**0.003**		
	4 cm vs 7 cm	4	176	0.55	0.879		
	Shallow vs Deep	4	1 448	4.07	**0.001**		
	Location(Season)	10	212			0	0.0
	Depth × Location(Season)	28	294			294	37.0
Pecks Cove	Season	2	1 119	2.28	0.078	55	12.2
	Depth	3	1 296	8.59	**0.001**	134	29.7
	Surface vs 1 cm	1	621	3.87	**0.025**		
	4 cm vs 7 cm	1	202	1.16	0.341		
	Shallow vs Deep	1	3 243	20.72	**0.001**		
	Season × Depth	6	212	1.40	0.108	21	4.7
	Location(Season)	6	493			89	19.9
	Depth × Location(Season)	17	151			151	33.5

Planned contrasts examined variations among depths or their seasonal patterns. Bold represents significant and interpretable *P* values of fixed effects. Estimates and percentages of variation are presented for all sources of variation. Negative estimates were replaced with zeros [48]. Number of unique permutations  = 818–999. PERMDISP tests for Grande Anse: *F*_19, 38_ = 1.77, *P* = 0.758; and Pecks Cove: *F*_3, 31_ = 0.77, *P* = 0.612.

**Fig. 3:**
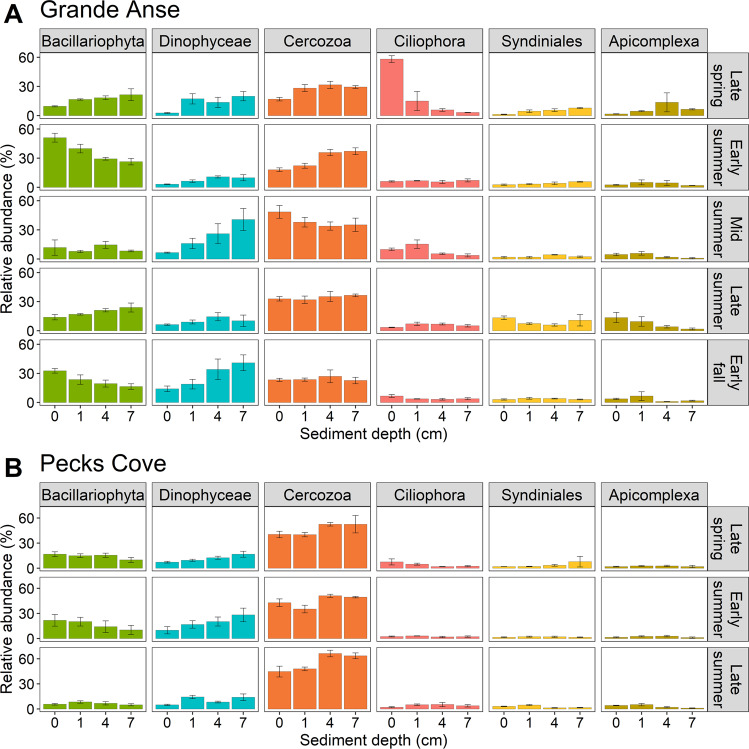
18S rDNA read abundance (mean ± SE) of dominant protist taxa among depths and seasons in Bay of Fundy mudflats in 2019. *n* = 3 samples, except in early summer (1 cm and 7 cm depths) at Grande Anse (**A**) and late summer (1 cm depth) at Pecks Cove (**B**) where *n* = 2.

The spatial and seasonal heterogeneity of protist communities was due mainly to consistent differences in the read abundances of Cercozoa, Bacillariophyta and Dinophyceae, the ‘core’ taxa in sediments (Table 2, Table S6). Ciliophora at Grande Anse and Vampyrellida at Pecks Cove were also important, albeit inconsistent, sources of variation. They, together with the ‘core’ taxa, were responsible for about two-thirds of community differences. Cercozoa was typically either more abundant in deep than shallow sediments on average, or it displayed no depth effect, while Bacillariophyta similarly showed different depth patterns depending on the season (Fig. 3). The composition of Bacillariophyta assemblages was depth-dependent at Grande Anse but not Pecks Cove. Specifically, at Grande Anse, raphid pennates generally decreased with depth, while polar centric Mediophyceae and araphid pennates were generally most abundant in deep sediments. Dinophyceae was generally more abundant in deep than shallow sediments and this was most apparent in mid summer and early fall (Fig. 3), while the opposite was true for Vampyrellida at Pecks Cove (Fig. S3). Ciliophora was highly abundant in late spring in Grande Anse surface sediments, a notable contrast to all other instances of its occurrence (Fig. 3). This can be singularly attributed to an individual ASV affiliated with *Zosterodasys* sp. (ASV1497, Table S3). It peaked to comprise 52% of protist reads on average but comprised just 0.4% of reads outside its peak occurrence and was prevalent in just 20% of samples. Interestingly, our preliminary exploration of the amplicon sequence data for Metazoa—which was dominated by nematode reads—revealed that metazoan read abundance was significantly associated with variations in the abundance of Apicomplexa reads, albeit seasonally. Other less prominent taxa were similarly dynamic but only marginally contributed to spatio-seasonal variations and community dissimilarity at either site (Figs. S3 and S4, Table S6).

**Table 2:** SIMPER results showing the contributions of protist taxa and trophic functional subgroups to significant variations among depths or their seasonal patterns (see Table 1 and S7) in Bay of Fundy mudflats in 2019.

Group	Overall average dissimilarity (%) and contributions to that dissimilarity (%)			
	Grande Anse	Grande Anse	Pecks Cove	Pecks Cove
	Surface vs 1 cm	Shallow vs Deep	Surface vs 1 cm	Shallow vs Deep
*Average dissimilarity*	*27.2*	*32.3*	*25.0*	*29.4*
Amoebozoa	1.9	**1.8**	**6.1**	**4.5**
Apicomplexa	8.3	8.5	**3.4**	**3.1**
Apusomonadidae	**4.5**	**3.5**	**6.3**	**6.5**
Bacillariophyta	**15.2**	**15.5**	**17.6**	**15.4**
Cercozoa	**14.3**	**15.2**	**16.6**	**26.4**
Chlorophyta	**3.8**	**3.3**	**3.9**	**3.6**
Ciliophora	22.3	15.5	5.7	4.4
Dinophyceae	**14.6**	**22.7**	**14.9**	**16.0**
Ichthyosporea	0.6	**0.6**	**1.2**	**0.8**
Labyrinthulomycetes	**0.8**	**0.8**	**1.2**	**1.2**
MAST-9	**0.9**	**0.8**	**1.3**	0.8
Other Endomyxa	1.1	0.7	**0.5**	**0.4**
Perkinsea	2.5	2.0	0.6	0.4
Syndiniales	**5.2**	**5.0**	**2.5**	3.8
Vampyrellida	**2.7**	**2.6**	16.2	11.0
*Average dissimilarity*	*23.8*	*26.1*		*21.3*
Phototroph	**22.2**	**28.6**		**22.9**
Eukaryvore	**7.9**	**8.2**		**27.7**
Bacterivore	**8.0**	**6.8**		**11.2**
Omnivore	24.3	17.7		6.4
Invertebrate parasite	**9.2**	11.7		5.0
Protist parasite	**5.9**	**6.3**		5.3

Top contributors are shown here; see Tables S6 and S10 for all contributors. Overall average community dissimilarity among depths or their seasonal patterns is italicized. Bold indicates taxa or trophic functional subgroups with consistent contributions to dissimilarity (i.e., average dissimilarity/SD of dissimilarities ≥ 1).

### Trophic functional perspective of community dynamics

Consumers dominated protist communities in sediments, accounting for 45% of reads on average and 48% of ASVs (Table S3). Among consumers, eukaryvores (consume eukaryotes) comprised 43% of reads, bacterivores (consume bacteria) and omnivores (consume eukaryotes and bacteria) each comprised 14%, and consumers of unknown prey (hereafter “unknown consumers”) comprised 30%. Eukaryvores were dominated by Cercozoa (75% of reads), bacterivores by Cercozoa (44%) and Apusomonadidae (41%), and omnivores by Ciliophora (97%). Phototrophs (exclusively photosynthetic lineages) represented 30% of protist reads and 12% of ASVs, while mixotrophs (utilizing both organic and inorganic sources of nutrition) comprised <1% of reads and ASVs (Table S3). Bacillariophyta (60%), Dinophyceae (25%) and Chlorophyta (14%) comprised most reads among phototrophs. Parasites accounted for 10% of protist reads and 13% of ASVs (Table S3). Invertebrate parasites (9% of protist ASVs) and protist parasites (3% of protist ASVs) were equally abundant, with each accounting for 49% of reads among parasites. The class Gregarinomorphea of Apicomplexa comprised 77% of reads among invertebrate parasites while the Group I Clade 4 lineage of Syndiniales comprised 81% of reads among protist parasites. Parasites of vertebrates, unknown animals, plants, and uncertain hosts were also observed. Nutritionally ambiguous (i.e., trophically unassignable) taxa notably comprised 15% of protist reads and 26% of ASVs (Table S3). Dinophyceae and Cercozoa together comprised 89% of these reads while Cercozoa alone comprised 70% of the ASVs. Simply put, while about half of reads among Dinophyceae were affiliated with phototrophs, a sizable minority (44% of reads) were nutritionally ambiguous.

The taxonomic and trophic functional perspectives of protist community dynamics were significantly correlated, although to a greater degree at Grande Anse (RELATE Spearman’s *ρ* = 0.76, Fig. 2A, B) than Pecks Cove (*ρ* = 0.55, Fig. 2C, D). The relative importance of each component of variation was also broadly consistent between these biotic perspectives (Table 1, Table S7). From an abiotic perspective, our measurements indicated that the pH, oxygen content and temperature of the sediment environment decreased progressively with depth and displayed seasonality (Fig. S5, Table S8). While significant variations in physico-chemical properties between shallow and deep sediments, coincided with similar variations in community composition (from both perspectives), these variables were weakly correlated (Table S9).

**Fig. 4:**
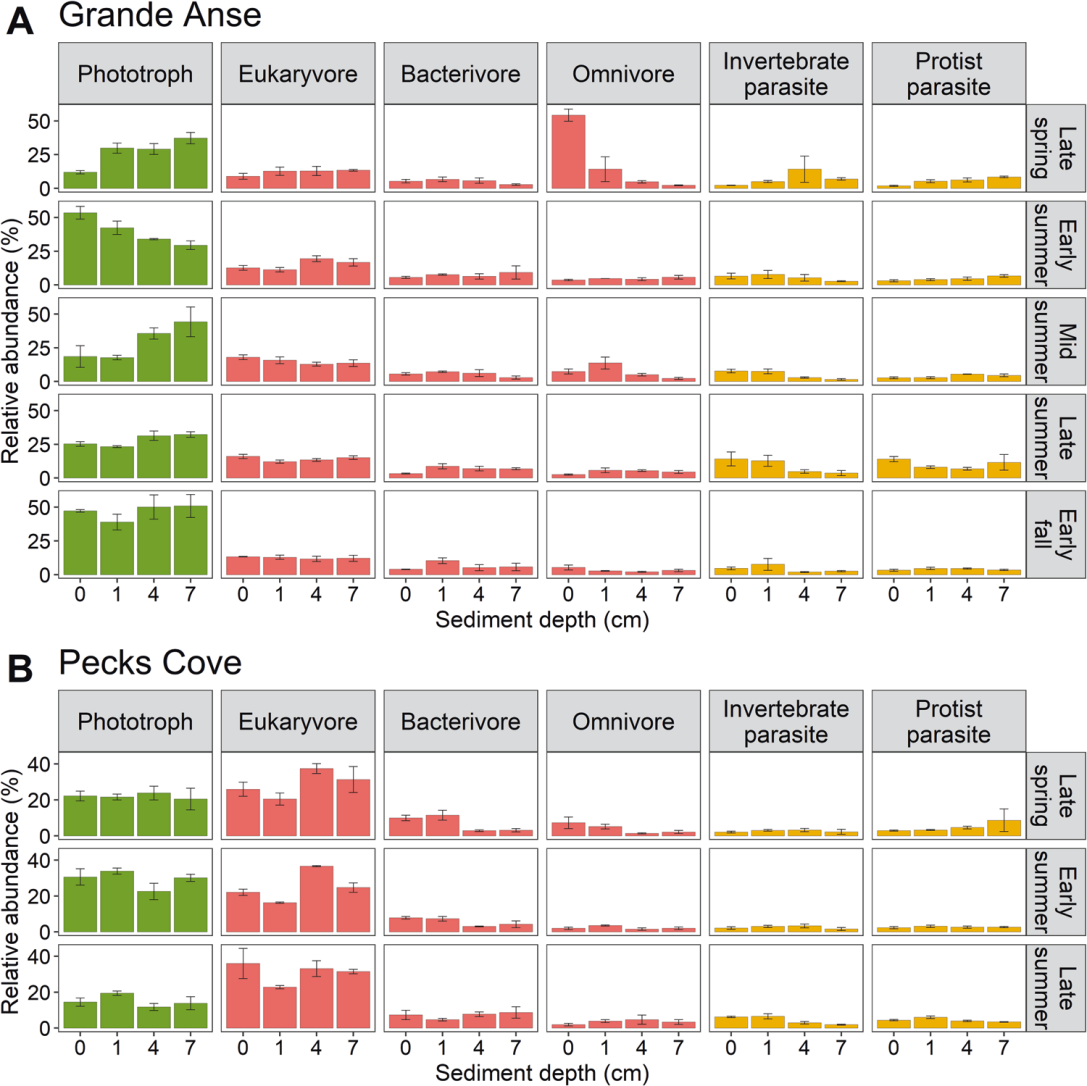
18S rDNA read abundance (mean ± SE) of dominant protist trophic functional subgroups among depths and seasons in Bay of Fundy mudflats in 2019. *n* = 3 samples, except in early summer (1 cm and 7 cm depths) at Grande Anse (**A**) and late summer (1 cm depth) at Pecks Cove (**B**) where *n* = 2. Abundant “Unknown consumer” and “Unknown nutrition” subgroups are not shown here. Less abundant “Mixotroph”, “Vertebrate parasite”, “Unknown animal parasite”, “Plant parasite”, and “Unknown parasite” subgroups are also not shown.

Omnivores and phototrophs were top contributors to community variations at Grande Anse and the same is true of eukaryvores and phototrophs at Pecks Cove (Table 2, Table S10). The dynamics of trophic functional subgroups broadly reflected their dominant constituent taxa (Fig. 4, Table S3). Consumers displayed complex dynamics across depths and seasons (Fig. 4). Among consumers, eukaryvores were on average twice as abundant at Pecks Cove than Grande Anse (28% vs 14% of reads) while bacterivores were equally abundant at both sites. Phototrophs peaked in early summer and early fall, while invertebrate and protist parasites both peaked in late summer, and this was most apparent in shallow sediments (Fig. 4). Parasites were also generally more abundant at Grande Anse than Pecks Cove.

## Discussion

### Dominant ‘SAR’ lineages constitute the core eukaryotic microbiome in sediments

We surveyed two mudflats in the upper Bay of Fundy (hereafter “Fundy sediments”) for insights into major food web players, diversity patterns and potential local- and regional-scale processes structuring hypertidal microbenthos. Regarding the major food web players, Cercozoa, Bacillariophyta and Dinophyceae (all members of the ‘SAR’ clade) were dominant across the spatial and temporal scales we evaluated—in terms of read abundance, ASV richness and prevalence—suggesting that they possess high dispersal capacity and other ecologically favourable traits. Our findings are consistent with estimates that the ‘SAR’ clade comprises most protist diversity [54]. At the coarse taxonomic scale examined, we therefore defined Cercozoa, Bacillariophyta and Dinophyceae as the core eukaryotic microbiome in Fundy sediments.

Cercozoa was the richest and most abundant taxon in Fundy sediments, consistent with findings from most major biomes and ecosystems [55]. Cercozoa was also the dominant consumer in both Fundy sediments and terrestrial soil, in contrast to marine plankton [55]. However, Cercozoa was more prominent in Fundy sediments than soil, with the opposite being true of consumers broadly [16, 56]. Although Cercozoa specifically dominated eukaryvorous consumers in Fundy sediments, it also expectedly maintained a degree of trophic functional versatility. The class Thecofilosea (characterized by robust extracellular theca [21]) was dominant among Cercozoa in Fundy sediments. This is consistent with ribosomally active protist diversity in subtidal sediments but differs from soil where naked cercozoans like glissomonads dominated [57–59]. Shells (theca and tests) may offer protection against predation, desiccation, and hydrological instability [15, 60]; all stressors that cercozoans are exposed to in Fundy sediments. Protaspa-lineage, a family of thecofilosean biflagellates [61, 62], was a dominant presence in Fundy sediments. Its members employ hydrodynamic flagella for substrate-mediated gliding and pseudopodia for phagocytosis of eukaryotic prey [61]. The filose amoeboid genus *Paulinella* includes photosynthetic species like P. *chromatophora*—which we detected in Fundy sediments—but also numerous non-photosynthetic representatives [63, 64]. Photosynthetic *Paulinella* species are important models for studying the endosymbiotic evolution of photosynthetic organelles and interestingly, P. *chromatophora* had previously only been reported from freshwater habitats [63, 65]. Most ASVs affiliated with *Paulinella* in Fundy sediments were taxonomically unresolved and consequently cannot be inferred to be photosynthetic; thus, further research is needed to elucidate their taxonomic identities, functional traits, and ecological roles.

Bacillariophyta (diatom) was notably less prominent in Fundy sediments than European coastal sediments where it accounted for 40% of protist reads in subtidal zones, twice its contribution in intertidal Fundy sediments [66]. Bacillariophyta was also the dominant phototroph in Fundy sediments and marine plankton, but not freshwater plankton [55, 59]. These microalgae are key producers of organic matter in coastal upwelling systems and important players in the marine biogeochemical cycling of carbon, silicon, and nitrogen [67]. The typically planktonic genus *Thalassiosira* dominated diatom assemblages in Fundy sediments, in contrast to European coastal sediments where other typically planktonic diatoms dominated [66]. Both these studies provide evidence of strong coupling between the plankton and benthos. Intriguingly, diatom read abundance was comparable between our shallowest and deepest Fundy sediments, possibly also due to strong coupling. The diatom DNA signatures detected in our deepest sediments may originate from resting stages (which can remain viable for millennia [68]), or vegetative cells. Planktonic diatoms in the North Pacific Ocean were found to be exported to aphotic and anoxic sediments as ribosomally active vegetative cells [59]. In addition, *Thalassiosira* and *Skeletonema* were found to dominate the eukaryotic metatranscriptome in aphotic and anoxic Baltic Sea sediments [69]. In the absence of light and oxygen, diatom species that store nitrate intracellularly (like some within *Thalassiosira*) can use it for dissimilatory nitrate reduction to ammonium, a form of anaerobic respiration [70, 71]. Broman et al. [69] found that while neither the read abundance nor mRNA profile (metabolic signature) of benthic diatoms is oxygen-sensitive, these attributes are responsive to light exposure, which could arise from regular resuspension (or other disturbances) in Fundy sediments. Further research using RNA-based tools (e.g., metatranscriptomics) is needed to elucidate the ecological role of diatoms in our deep Fundy sediments.

Dinophyceae (core dinoflagellate) was relatively less rich in Fundy sediments than ocean plankton where it accounted for half of the protist richness [72]. While the order Gonyaulacales had a dominant presence in both ecosystems, the genus *Alexandrium* was most abundant in Fundy sediments, with *Ceratium* and *Gonyaulax* featuring prominently in global ocean plankton [72]. *Alexandrium* species are trophically opportunistic and capable of forming non-motile cysts in response to environmental stress (e.g., turbulence, parasitism, grazing and nutrient depletion) [51]. There is evidence of widespread accumulation of *Alexandrium* cysts in outer Bay of Fundy sediments, likely from successive cyst depositions after blooms [73, 74]. Given the strong tidal currents in this region, the same may be true of the upper Bay of Fundy where our sites lie. Thus, Fundy sediments may constitute a repository of genetic and functional microbial diversity, as suggested for European coastal sediments [75]. The potential for hydrodynamic transport of toxigenic, bloom-forming *Alexandrium* from Fundy sediments back into the water column is concerning for human and ecosystem health. The abundant 18S rDNA reads affiliated with *Alexandrium fundyense*, as well as our PCR detection of the *sxtA4* gene—which is specific to saxitoxin producers [76]—provide evidence of the presence of toxigenic dinoflagellates in Fundy sediments. While our metabarcoding approach was limited in distinguishing active vegetative cells from dormant cysts, metagenomic and metatranscriptomic surveys would offer greater resolution and insight into dinoflagellate assemblages.

The ‘core’ taxa in Fundy sediments were typically well represented in most ecosystems including marine sediments [19, 59, 75, 77–79], ocean plankton [18, 80, 81], soil [16, 56, 57] and freshwater [82–84], although discrepancies existed. Specifically, in comparison to Fundy sediments, different groups were most abundant in deep-sea sediments and plankton. Also, Retaria was the most abundant rhizarian lineage in photic zone ocean plankton, compared to Cercozoa in Fundy sediments. Lastly, chrysophytes were poorly represented in Fundy sediments but (alongside ciliates) dominated benthic and planktonic communities in lakes. In European coastal sediments, Ciliophora and Amoebozoa were underrepresented while Apicomplexa and Dinophyceae were overrepresented in DNA-based (compared to RNA-based) surveys [19]. This highlights the ambiguity surrounding the active role of dinoflagellates in marine sediments, including ours, and suggests that Ciliophora may play a more prominent role in Fundy sediments than our read abundance estimates suggest. Conversely, the occurrence of polyploid macronucleus in ciliates, as well as the high copy number and intra-individual polymorphisms of the 18S rRNA gene in this lineage, complicates our abundance and richness estimations, and may inflate estimates for ciliates [41, 85]. The high copy number of nuclear genes in dinoflagellates similarly complicates abundance estimations using amplicon-based (DNA metabarcoding) approaches [86, 87].

The relative importance of each trophic functional group in ecosystem functioning differed between Fundy sediments and other ecosystems, although consistencies existed. Consumers were much richer and abundant than phototrophs or parasites in Fundy sediments, like most ecosystems [16, 18, 55]. Phototrophs comprised almost a third of protist reads in Fundy sediments, which contrasts with its much higher read abundance in arid soil and freshwater plankton [16, 55]. Parasites represented 10% of protist reads in Fundy sediments, midway between freshwater plankton (5%), and marine plankton, subtidal sediments, and soil (15–20%) [55]. Syndiniales (with its parasitoid life-history strategy) overwhelmingly dominates parasite assemblages in marine plankton, while Apicomplexa is hyperdiverse and dominant in soil ecosystems (particularly neotropical rainforests) [16, 25, 55, 57]. However, parasite assemblages in Fundy sediments neither mirrored soil nor marine plankton, instead, Syndiniales and Apicomplexa were equally abundant. Interestingly, the richness of Apicomplexa in Fundy sediments is comparable to reports from Arctic sandy tidal-flat sediments [88]. Apicomplexa was considerably (6×) richer than Syndiniales, possibly due to the abundant potential faunal hosts in Fundy sediments and host specificity within this taxon. Most ASVs among Apicomplexa grouped with gregarines, like in neotropical rainforests [25]. Gregarines are known to exclusively infect invertebrates, except for a single reported case in a vertebrate host [89, 90]. Host density is a reliable driver of parasite dynamics and as such, apicomplexan dynamics in Fundy sediments may parallel its infaunal hosts, which are much better studied [6, 32]. Syndiniales were about twice as abundant, among parasites, in Fundy than subtidal sediments [78]. The broad host range and life history (abundant progeny) of Syndiniales likely contributed to its prominence [91]. While Syndiniales may play an important role in regulating the abundances of other microbes in Fundy sediments, Apicomplexa may be a key facilitator of macro-/micro-organismal interactions. These findings likely reflect the situation of intertidal mudflats near the intersection of terrestrial and marine ecosystems.

### Interplay between local and regional processes may drive heterogenous microbial distribution

Protist communities in Fundy sediments were heterogeneous across the spatial and seasonal scales evaluated. This community heterogeneity is reflective of the inherent dynamicity of the intertidal sediment environment, in contrast to the relatively buffered conditions of overlying waters [2]. Major factors underlying environmental dynamicity include cyclical tidal action, variable sedimentation regimes, bioturbation and bio-irrigation activities of ecosystem-engineering invertebrates, and migratory shorebird presence [1, 92, 93]. Environmental selection has been found to influence protist communities in coastal sediments [78, 79]; however, these relationships were weak and inconsistent in Fundy sediments. Site-specific factors such as the major freshwater input (Petitcodiac River) at Grande Anse and relatively higher densities of predatory infauna at Pecks Cove may have contributed to the more pronounced  community heterogeneity at Grande Anse. Water content is widely understood to be strongly associated with protist community composition in soil [16, 56, 94] and the same may be true in Fundy sediments given the lesser sediment porewater (and nutrient) content at Pecks Cove than Grande Anse [6, 14]. Protist communities in Fundy sediments were highly variable at the sediment surface (generally more so than other depths) likely owing to their relatively greater exposure and susceptibility to cyclical environmental change, and disturbance. The trophic functional dynamics of protists in Fundy sediments mirrored the one or two dominant taxa in each subgroup, leading us to question how functionally redundant and resilient these protist communities are [95], thus requiring further research.

The increased potential for benthic-pelagic coupling (i.e., nutrient and biomass exchange) in our hypertidal Fundy mudflats sets them apart from most other marine sedimentary ecosystems. For instance, coastal plankton were found to have low genetic similarity with the subtidal benthos in Europe but similar isotopic signatures with the intertidal benthos in the Bay of Fundy [75, 96]. 72% of protist ASVs in Fundy sediments were unexpectedly present at both sites, given the strong site-level variations observed in protist community composition. These sites were 6 km apart overland and 21 km apart along the coastline. In contrast, two sandy intertidal beaches 5.6 km apart in Connecticut, USA shared just 32% of operational taxonomic units [79]. Consequently, microbial genetic diversity in Bay of Fundy sediments may follow a fairly uniform distribution, in line with the ‘cosmopolitan model’ of microbial biogeography [97] and contrasting the ‘endemicity model’ [98]. Protist communities in Fundy sediments also varied substantially at the level of individual samples reflecting the high degree of patchiness in this system. Complex community dynamics can arise from synergistic interactions of several factors including environmental selection, biotic interactions, dispersal limitation, and ecological drift [30, 31]. However, the relative importance of each can often vary between study sites and ecosystems. In Fundy sediments, a possible explanation for the high genetic linkages, and strong site- and sample-level variability is the coalescence of (i) a regional species pool stochastically governing near uniform diversity distribution in the Bay of Fundy, (ii) site-specific environmental regimes partially regulating species abundance, and (iii) complex species interactions and co-occurrence patterns creating patchiness. While we did not examine patterns of rare ASVs, their high richness (14,599 ASVs or 83% of the pre-filtration total, Table S2) and presumed limited dispersal would likely contribute to community differences [79]. Further investigation is needed to elucidate the ecological role of the rare biosphere where the bulk of protist diversity exists (this study, [99])—including through the use of enrichment culture approaches [100]—and resolve the identities of unclassified ASVs (at the phylum-level) which we discarded as part of our quality control protocol (Table S2).

## Conclusion

We quantified spatio-seasonal dynamics and inventoried protist diversity in intertidal mudflats, an ecosystem characterized by harsh conditions. Protist diversity in Fundy sediments were previously unexplored at the community-wide scale, and to the best of our knowledge, our survey is the first of its kind in a hypertidal benthic ecosystem. We defined the core eukaryotic microbiome in Fundy sediments, which while being consistently dominant, also contributed most to overall community variations. Further research is needed to explore the relative contributions of various deterministic and stochastic processes to microbial community assembly in Bay of Fundy mudflats, including selection, dispersal, and ecological drift [30, 31], as well as the potential role of benthic-pelagic coupling as a key driver of microbial community dynamics in high-energy coastal systems. Future studies should also examine potential interactions within the sediment microbiome using co-occurrence networks [101], as well as investigate trophic linkages between microbial components of Fundy sediments and both the ecosystem-engineering infaunal residents and epibenthic visitors. Our intriguing detection of relatively abundant diatom and dinoflagellate assemblages at depth necessitates RNA-based examination of their activity state and potential ecological roles. Our study contributes to the broader understanding of processes structuring microbial communities in diverse environments and begins to integrate mudflat protists into the broader framework of marine microbiome research.

## Supplementary information

Supplementary Information

Table S3

Table S6

Table S10

## Data Availability

The amplicon sequence datasets generated and analyzed during the current study are publicly available in the NCBI Sequence Read Archive (SRA) repository under accession number PRJNA932559.
