# Supplementary material for: Community dynamics of microbial eukaryotes in intertidal mudflats in the hypertidal Bay of Fundy
Source: ISME Commun. 2023 Mar 14;3:21. doi: 10.1038/s43705-023-00226-8 (PMC10014957; doi:10.1038/s43705-023-00226-8)
Supplement: Supplementary file 1 — Supplementary Information [file 43705_2023_226_MOESM1_ESM.pdf]

Supplementary Information for: Community dynamics of microbial eukaryotes in intertidal mudflats in the hypertidal Bay of Fundy

Eke I. Kalu, Adrian Reyes-Prieto, Myriam A. Barbeau

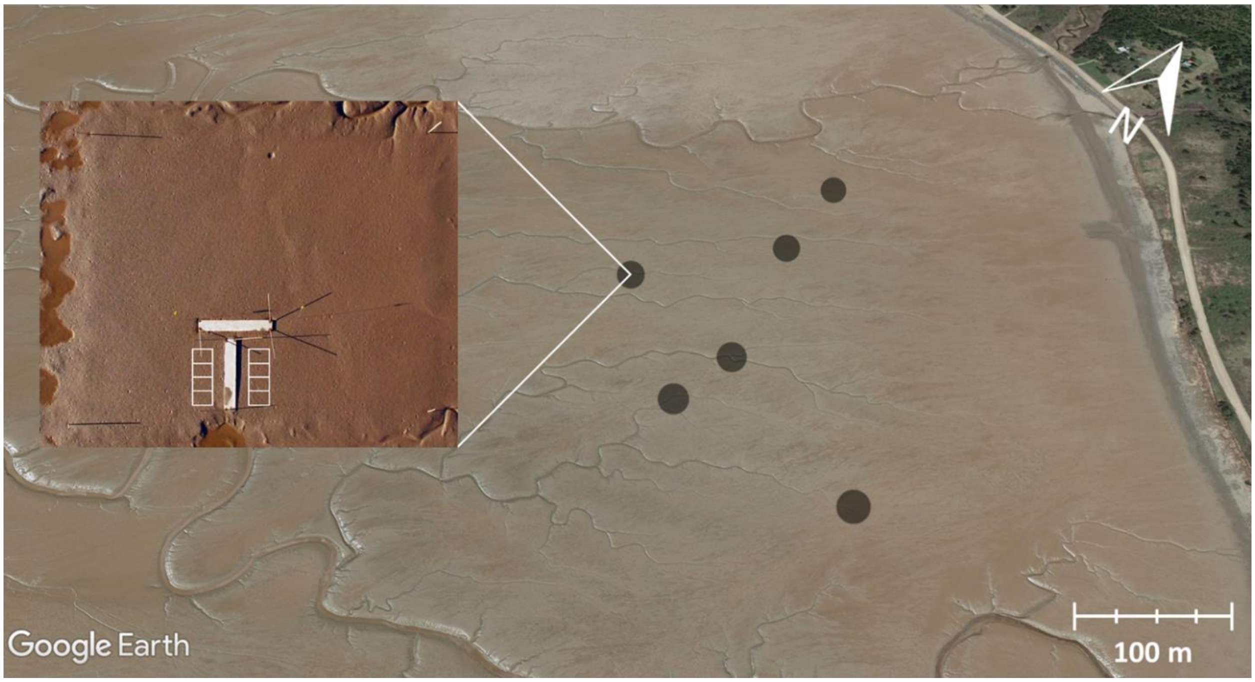

Figure S1. Schematic of the study area at Grande Anse showing the layout of six 5 m x 6 m plots (dark circles) and inserted aerial view of one such plot showing the layout of the eight 0.35 m x 0.5 m sampling locations delineated along a planked walkway. Individual plots and sampling locations were set up far enough from one another to be independent measures of sediment biotic and abiotic properties. Photograph taken by G.S. Norris in summer 2019.

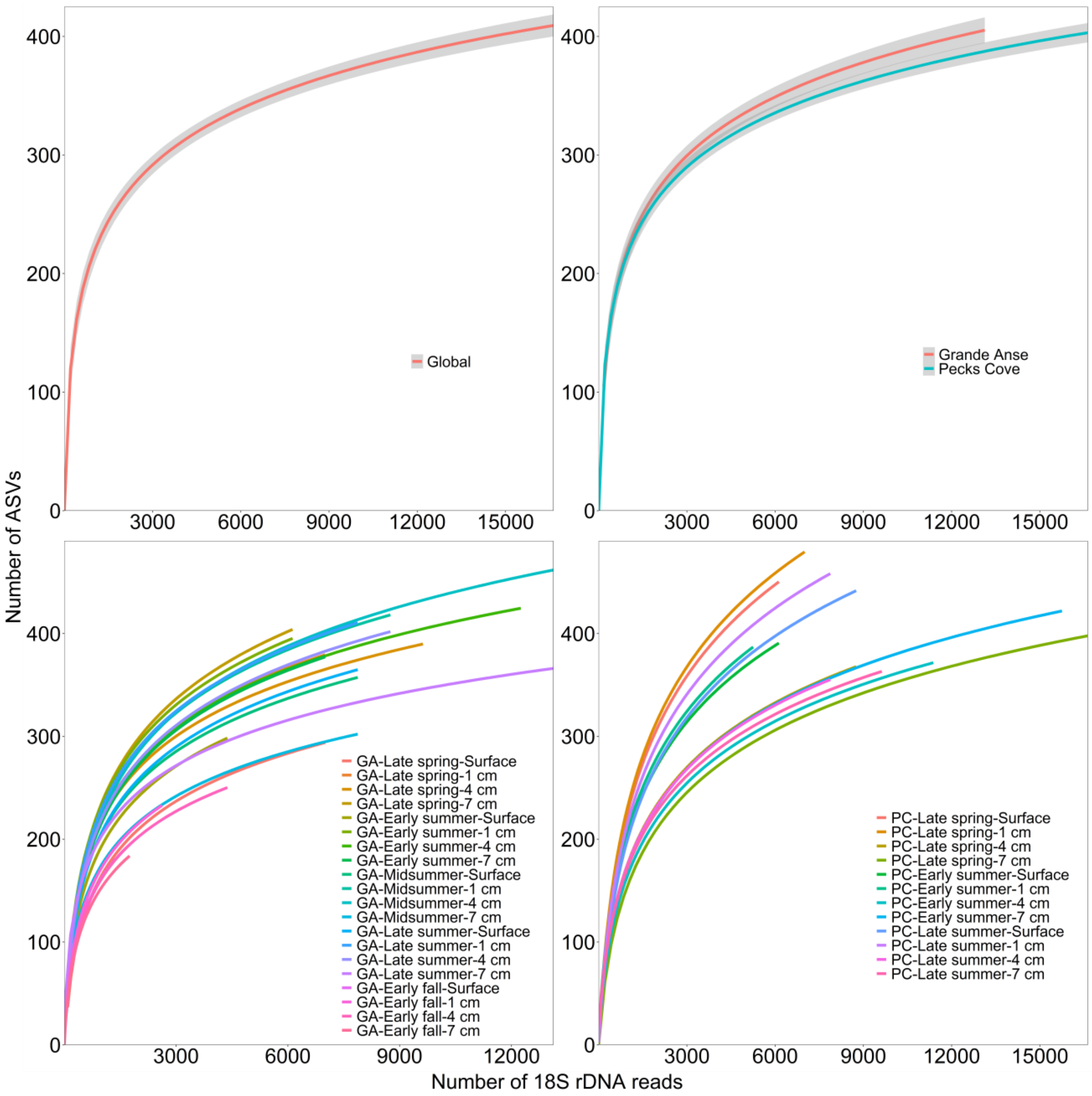

Figure S2. Global and local (i.e., each site and combination of site, season, and depth) rarefaction curves of protist ASVs in Bay of Fundy mudflats in 2019. Logarithmic trendlines are shown for all curves. 95% confidence intervals are shown for global and site-level curves. GA, Grande Anse. PC, Pecks Cove. n = 93, 58 and 35 samples per curve for the global, Grande Anse-level, and Pecks Cove-level analyses, respectively. n = 3 samples per curve for the site-season-depth combination analyses, except for early summer (1 cm and 7 cm depths) at Grande Anse and late summer (1 cm depth) at Pecks Cove where n = 2.

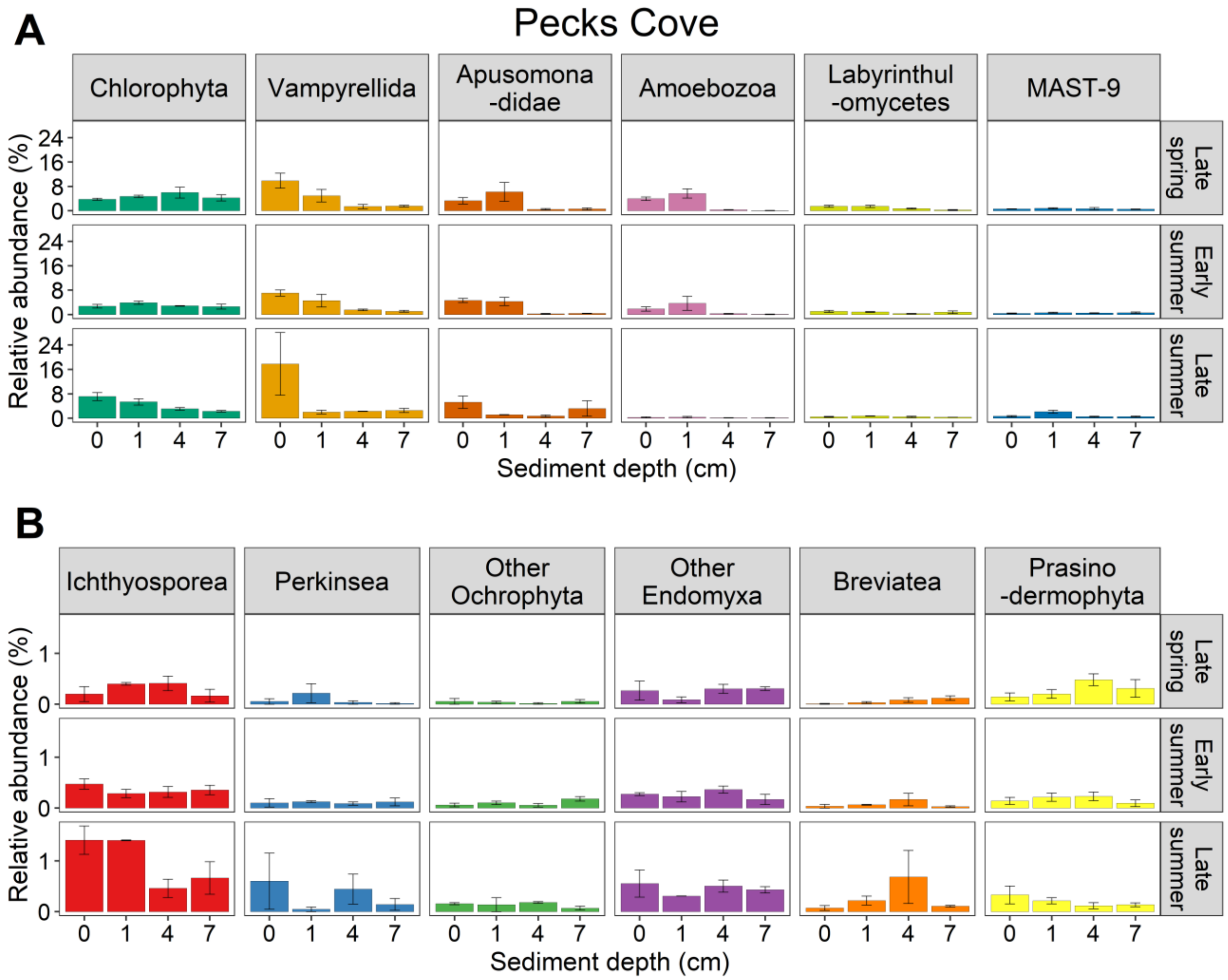

Figure S3. 18S rDNA read abundance (mean ± SE) of protist taxa among depths and seasons at Pecks Cove in 2019. Relatively less abundant taxa are shown here; see Figure 3 for dominant taxa. n = 3 samples, except in late summer where n = 2 for the 1 cm depth. Taxa with average read abundances of <0.1% are not shown here; these include Choanoflagellida, Telonemia, Centroheliozoa, Katablepharidophyta, MAST-6, Cryptophyta, Discoba, Streptophyta, Chrompodellids, Haptophyta, Rhodophyta, Radiolaria, Planomonadida and Hemimastigophora (in order of decreasing abundance). "Other Endomyxa" are non-Vampyrellida endomyxans. "Other Ochrophyta" are non-Bacillariophyta ochrophytes.

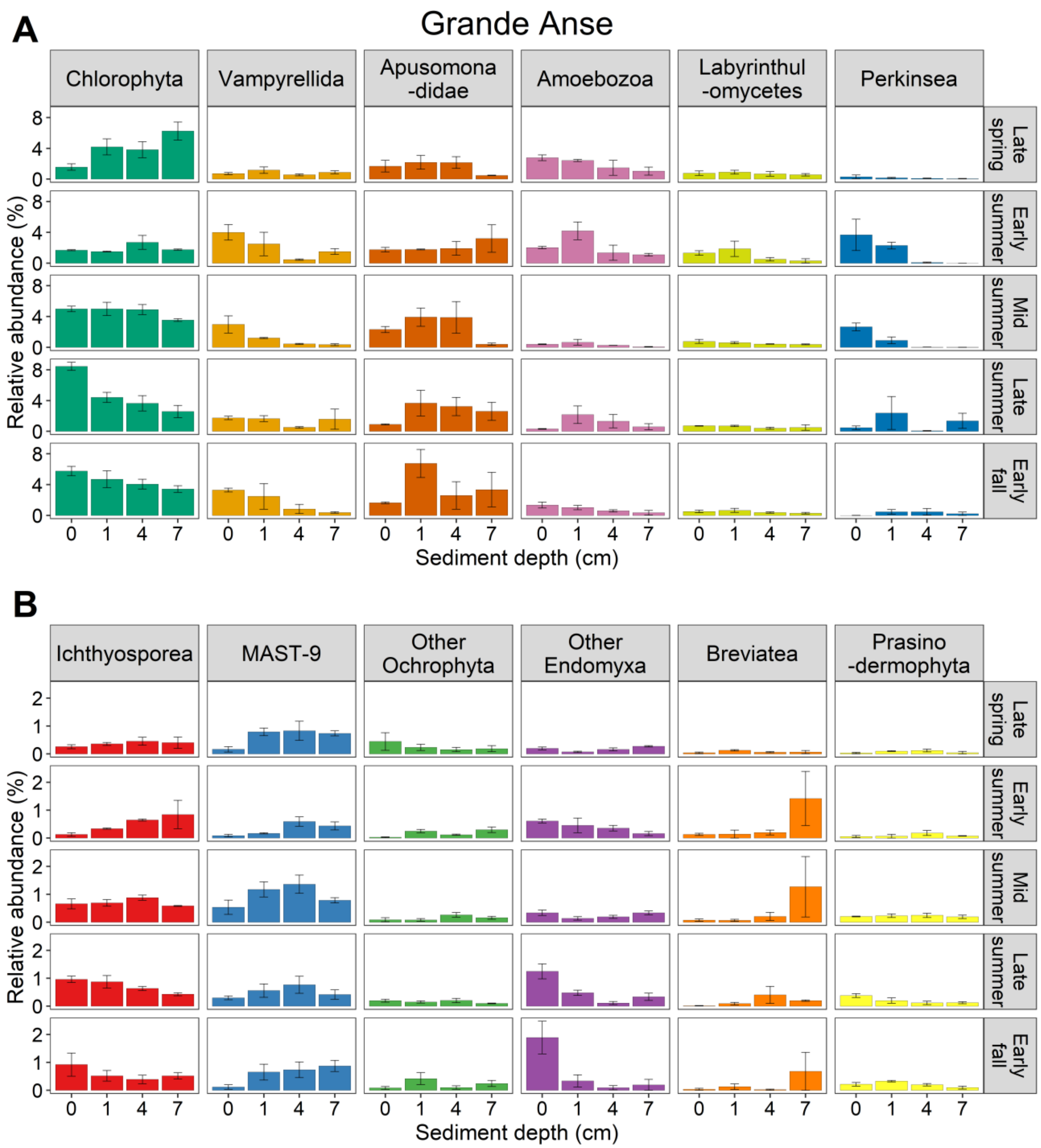

Figure S4. 18S rDNA read abundance (mean ± SE) of protist taxa among depths and seasons at Grande Anse in 2019. Relatively less abundant taxa are shown here; see Figure 3 for dominant taxa. n = 3 samples, except in early summer where n = 2 for the 1 cm and 7 cm depths. Taxa with average read abundances of <0.1% are not shown here; these include Choanoflagellida, Telonemia, Centroheliozoa, Katablepharidophyta, MAST-6, Cryptophyta, Discoba, Streptophyta, Chrompodellids, Haptophyta, Rhodophyta, Radiolaria, Planomonadida and Hemimastigophora (in order of decreasing abundance). "Other Endomyxa" are non-Vampyrellida endomyxans. "Other Ochrophyta" are non-Bacillariophyta ochrophytes.

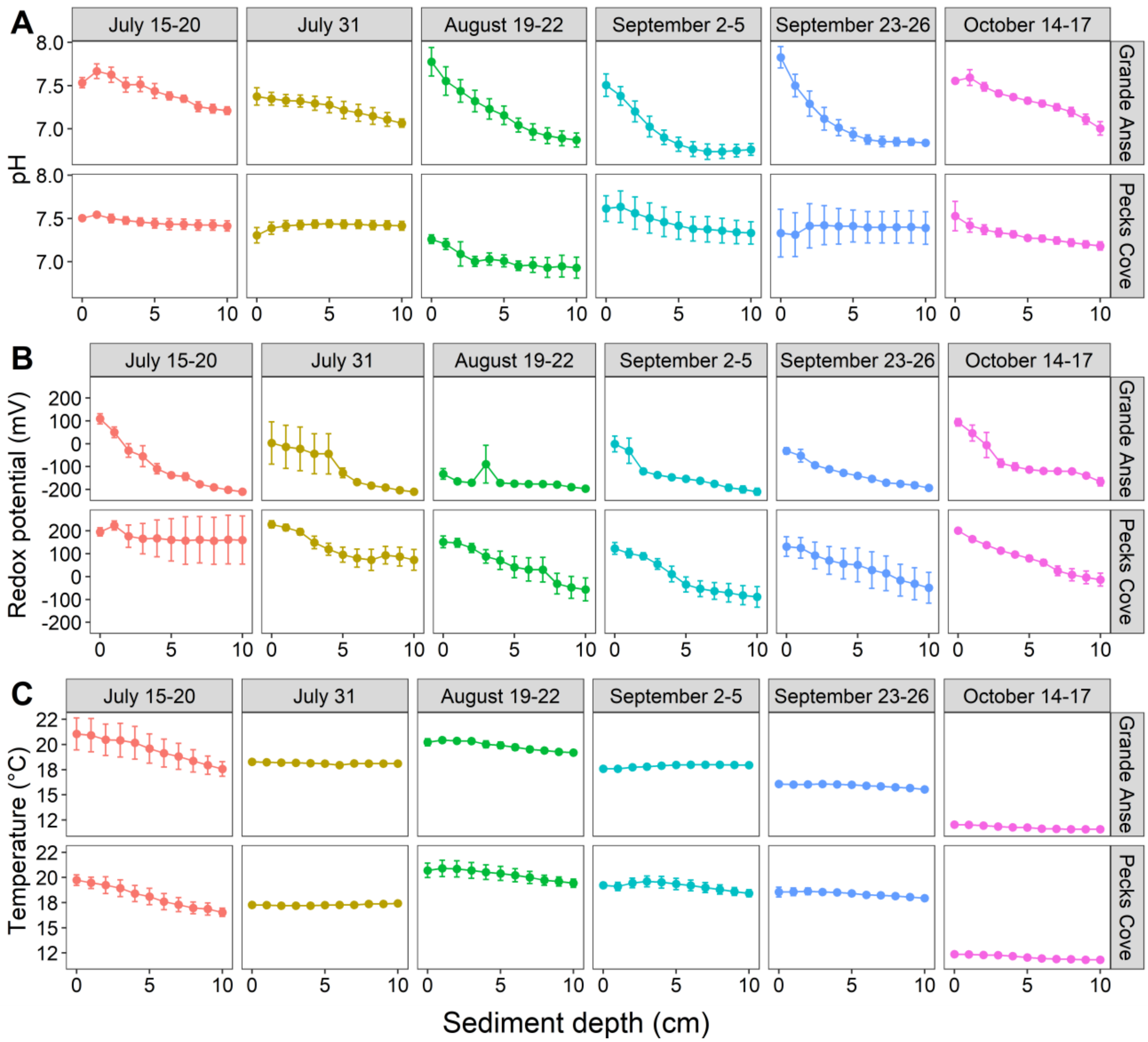

Figure S5. Depth profile (mean ± SE) of sediment (A) pH, (B) redox potential and (C) temperature over time in Bay of Fundy mudflats in 2019. n = 4 locations, except in July 15-20 at Pecks Cove where n = 3.

Supplementary Information

Table S1. Sampling dates for Bay of Fundy mudflats in 2019 and corresponding natural disturbances.

Season	Date		Natural disturbance
	Grande Anse	Pecks Cove	
Late spring	June 8	June 3	After winter disturbance
Early summer	July 20	July 15	Before shorebird arrival
Mid summer	July 31	July 31	High shorebird density
Mid summer	August 19*	August 22*	
Late summer	September 2	September 5	After shorebird departure
Early fall	September 23*	September 26*	
Early fall	October 14	October 17	Before winter disturbance

Community sampling corresponded with natural disturbances. Sediment physico-chemical properties were not measured in June, but measurements (*) were obtained in August and September without corresponding community sampling. Community data from mid summer and early fall at Pecks Cove were subsequently discarded as part of the bioinformatics quality control process.

Supplementary Information

Table S2. Counts and percentages for different categories of 18S rDNA reads and amplicon sequence variants (ASVs) that were discarded or retained during the bioinformatics component of our survey of Bay of Fundy mudflats in 2019.

Classification	Reads		ASVs	
	Count	%	Count	%
Mismatched primers	88 412	4.1		
Unjoined paired-end reads	211 132	9.8		
Low Phred quality score	421	0.0		
Erroneous or chimeric	673 216	31.2		
Rare ASV (<0.1% of reads)	37 506	1.7	14 599	83.4
Unclassified ASV (at the phylum level)	13 447	0.6	200	1.1
Metazoa	505 654	23.4	163	0.9
Fungi	43 607	2.0	381	2.2
Embryophyta (land plant)	1 566	0.1	23	0.1
Low read count sample	21 539	1.0	21	0.1
Retained (protist)	562 941	26.1	2 118	12.1

562 941 of 2 159 441 reads, 2 118 of 17 505 ASVs and 93 of 120 samples were retained.

Supplementary Information

Table S3. Annotation of protist ASVs (.xlsx file)

Table S4. PERMANOVA results for spatio-seasonal variations in protist richness (number of ASVs) in Bay of Fundy mudflats in 2019.

Site	Source of variation	df	MS	Pseudo-*F*	*P*	Component of variation Estimate	%
Both	Site	1	16 665	1.79	0.205		
	Season	2	4 878	0.52	0.607		
	Depth	3	9 844	1.20	0.316		
	Site x Season	2	6 953	0.75	0.490		
	Site x Depth	3	12 710	1.55	0.236		
	Season x Depth	6	5 960	0.73	0.652		
	Site x Season x Depth	6	1 526	0.19	0.977		
	Location(Site x Season)	12	9 302				
	Depth x Location(Site x Season)	33	8 178				
Grande Anse	Season	4	57 236	10.16	**0.007**	4 511	45.8
	Depth	3	12 865	2.90	0.062	601	6.1
	Season x Depth	12	4 192	0.95	0.495	0	0.0
	Location(Season)	10	5 612			311	3.2
	Depth x Location(Season)	28	4 430			4 430	45.0
Pecks Cove	Season	2	1 388	0.12	0.852		
	Depth	3	6 962	0.68	0.567		
	Season x Depth	6	3 801	0.37	0.883		
	Location(Season)	6	11 500				
	Depth x Location(Season)	17	10 276				

Bold represents significant and interpretable *P*-values of fixed effects. Estimates and percentages of variation are presented for all sources of variation, for analyses that showed significant patterns. Negative estimates were replaced with zeros (Searle *et al*. 1992). Number of unique permutations = 824-999.

Table S5. PERMANOVA results for spatio-seasonal variations in protist read count (number of 18S rDNA reads) in Bay of Fundy mudflats in 2019.

Site	Source of variation	df	MS	Pseudo-*F*	*P*	Component of variation Estimate	%
Both	Site	1	4 787 400	0.53	0.469		
	Season	2	6 260 600	0.69	0.552		
	Depth	3	36 016 000	2.72	0.072		
	Site x Season	2	18 135 000	2.00	0.167		
	Site x Depth	3	1 777 800	0.13	0.947		
	Season x Depth	6	6 092 400	0.46	0.860		
	Site x Season x Depth	6	4 728 400	0.36	0.904		
	Location(Site x Season)	12	9 052 900				
	Depth x Location(Site x Season)	33	13 221 000				
Grande Anse	Season	4	52 775 000	7.05	**0.012**	3 959 700	37.0
	Depth	3	18 855 000	3.64	**0.017**	974 420	9.1
	Surface vs 1 cm	1	2 246 900	0.44	0.506		0.0
	4 cm vs 7 cm	1	17 468 000	2.24	0.173		0.0
	Shallow vs Deep	1	37 570 000	7.36	**0.014**		0.0
	Season x Depth	12	4 295 000	0.83	0.619	0	0.0
	Location(Season)	10	7 459 900			601 620	5.6
	Depth x Location(Season)	28	5 173 800			5 173 800	48.3
Pecks Cove	Season	2	2 930 100	0.21	0.816		
	Depth	3	18 626 000	0.96	0.454		
	Season x Depth	6	5 201 100	0.27	0.952		
	Location(Season)	6	14 165 000				
	Depth x Location(Season)	17	19 380 000				

Planned contrasts examined variations among depths or their seasonal patterns. Bold represents significant and interpretable *P*-values of fixed effects. Estimates and percentages of variation are presented for all sources of variation, for analyses that showed significant patterns. Negative estimates were replaced with zeros (Searle *et al*. 1992). Number of unique permutations = 832-999.

Supplementary Information

Table S6. SIMPER results showing the contributions of protist taxa to community variations (.xlsx file)

Table S7. PERMANOVA results for spatio-seasonal variations in protist trophic functional subgroups in Bay of Fundy mudflats in 2019.

Site	Source of variation	df	MS	Pseudo-*F*	*P*	Component of variation Estimate	%
Both	Site	1	6 796	38.74	0.001	198	28.2
	Season	2	1 365	7.78	0.001	53	7.6
	Depth	3	784	5.06	0.001	38	5.4
	Site x Season	2	555	3.16	0.001	34	4.8
	Site x Depth	3	436	2.81	0.004	34	4.8
	Season x Depth	6	706	4.56	0.001	99	14.1
	Site x Season x Depth	6	394	2.54	**0.001**	86	12.2
	Location(Site x Season)	12	176			5	0.8
	Depth x Location(Site x Season)	33	155			155	22.1
Grande Anse	Season	4	1 400	8.65	0.001	108	21.0
	Depth	3	861	4.12	0.001	46	9.0
	Season x Depth	12	644	3.08	**0.001**	152	29.5
	Surface vs 1 cm	4	658	3.74	**0.003**		
	4 cm vs 7 cm	4	118	0.47	0.944		
	Shallow vs Deep	4	1 147	4.38	**0.001**		
	Location(Season)	10	161			0	0.0
	Depth x Location(Season)	28	209			209	40.5
Pecks Cove	Season	2	738	4.50	**0.018**	50	19.8
	Depth	3	471	3.12	**0.006**	37	14.7
	Surface vs 1 cm	1	303	3.38	0.080		
	4 cm vs 7 cm	1	322	1.57	0.221		
	Shallow vs Deep	1	800	5.41	**0.005**		
	Season x Depth	6	184	1.22	0.261	12	4.6
	Location(Season)	6	164			3	1.4
	Depth x Location(Season)	17	151			151	59.5

Planned contrasts examined variations among depths or their seasonal patterns. Bold represents significant and interpretable *P*-values of fixed effects. Estimates and percentages of variation are presented for all sources of variation. Negative estimates were replaced with zeros (Searle *et al.* 1992). Number of unique permutations = 828-999. PERMDISP tests for Grande Anse: $F_{19, 38}$=1.77, P=0.817; and Pecks Cove, $F_{3, 31}$=1.18, P=0.435.

Table S8. PERMANOVA results for spatio-temporal variations in sediment physico-chemical properties in Bay of Fundy mudflats in 2019.

Site	Source of variation	df	MS	Pseudo-*F*	*P*	Component of variation Estimate	%
Both	Site	1	274	35.38	0.001	1.04	25.4
	Date	5	103	13.32	0.001	1.11	27.2
	Depth	10	19	73.73	0.001	0.40	9.8
	Site x Date	5	21	2.73	0.005	0.31	7.6
	Site x Depth	10	4	13.85	0.001	0.14	3.4
	Date x Depth	50	1	2.80	0.001	0.06	1.4
	Site x Date x Depth	50	1	2.31	**0.001**	0.09	2.1
	Location(Site x Date)	35	8			0.68	16.7
	Depth x Location(Site x Date)	350	0			0.26	6.3
Grande Anse	Date	5	62	10.83	0.001	1.28	38.1
	Depth	10	23	52.17	0.001	0.96	28.6
	Date x Depth	50	1	2.68	**0.001**	0.19	5.6
	Surface vs 1 cm	5	1	1.47	0.204		
	4 cm vs 7 cm	5	1	1.54	0.157		
	Shallow vs Deep	5	5	7.68	**0.001**		
	Location(Date)	18	6			0.48	14.3
	Depth x Location(Date)	180	0			0.45	13.4
Pecks Cove	Date	5	72	5.20	0.001	1.39	42.0
	Depth	10	7	22.31	0.001	0.31	9.3
	Date x Depth	50	1	1.57	**0.002**	0.05	1.5
	Surface vs 1 cm	5	0	2.56	**0.033**		
	4 cm vs 7 cm	5	0	1.66	0.134		
	Shallow vs Deep	5	2	3.77	**0.001**		
	Location(Date)	17	14			1.23	37.3
	Depth x Location(Date)	170	0			0.33	9.9

Six dates (four across summer and two in early fall), 10 depths (surface to 10 cm) and 3-4 locations per site, date and depth, were assessed. Planned contrasts examined variations among depths or their temporal patterns. Bold represents significant and interpretable *P*-values of fixed effects. Estimates and percentages of variation are presented for all sources of variation. Number of unique permutations = 995-999.

Table S9. RELATE and BEST results showing associations between sediment physico-chemical properties and protist community composition in Bay of Fundy mudflats in 2019.

Site	Perspective	Property	RELATE		BEST
			Spearman's ρ	P	Spearman's ρ
Grande Anse	Taxonomy		0.19	**0.002**	
		pH			0.03
		Redox			0.21
		Temp			0.13
		pH, Redox			0.16
		pH, Temp			0.12
		Redox, Temp			0.22
		pH, Redox, Temp			0.19
Grande Anse	Trophic function		0.07	0.131	
Pecks Cove	Taxonomy		0.17	0.089	
Pecks Cove	Trophic function		0.11	0.109	

Redox, Redox potential. Temp, Temperature. Bold represents significant *P*-value. Number of samples across both sites = 69. Number of unique permutations = 999.

Table S10. SIMPER results showing the contributions of protist trophic functional subgroups to community variations (.xlsx file)

Supplementary Information

Reference

Searle SR, Casella G, McCulloch CE. Variance components. 1992. Wiley, New York, USA.
